# Genome-Wide Analysis of the Shi-Related Sequence Family and Functional Identification of *GmSRS18* Involving in Drought and Salt Stresses in Soybean

**DOI:** 10.3390/ijms21051810

**Published:** 2020-03-06

**Authors:** Shu-Ping Zhao, Xin-Yuan Song, Lin-Lin Guo, Xiang-Zhan Zhang, Wei-Jun Zheng

**Affiliations:** 1 College of Agronomy, Northwest A&F University/State Key Laboratory of Crop Stress Biology for Arid Areas, Yangling 712100, China; zhaoshuping001@163.com (S.-P.Z.); zhangxiangzhan2008@163.com (X.-Z.Z.); 2 Agro-biotechnology Research Institute, Jilin Academy of Agriculture Sciences, Changchun 130033, China; songxinyuan1980@163.com

**Keywords:** abiotic stress response, Genome-wide analysis, Negatively regulation, SRS family, Soybean

## Abstract

The plant-special *SHI-RELATED SEQUENCE* (SRS) family plays vital roles in various biological processes. However, the genome-wide analysis and abiotic stress-related functions of this family were less reported in soybean. In this work, 21 members of soybean SRS family were identified, which were divided into three groups (Group I, II, and III). The chromosome location and gene structure were analyzed, which indicated that the members in the same group may have similar functions. The analysis of stress-related *cis*-elements showed that the SRS family may be involved in abiotic stress signaling pathway. The analysis of expression patterns in various tissues demonstrated that SRS family may play crucial roles in special tissue-dependent regulatory networks. The data based on soybean RNA sequencing (RNA-seq) and quantitative Real-Time PCR (qRT-PCR) proved that SRS genes were induced by drought, NaCl, and exogenous abscisic acid (ABA). *GmSRS18* significantly induced by drought and NaCl was selected for further functional verification. *GmSRS18*, encoding a cell nuclear protein, could negatively regulate drought and salt resistance in transgenic *Arabidopsis*. It can affect stress-related physiological index, including chlorophyll, proline, and relative electrolyte leakage. Additionally, it inhibited the expression levels of stress-related marker genes. Taken together, these results provide valuable information for understanding the classification of soybean SRS transcription factors and indicates that SRS plays important roles in abiotic stress responses.

## 1. Introduction

The members of SHI-RELATED SEQUENCE (SRS) transcription factor family as the plant-specific family play crucial roles in plant growth and development. *LRP1* was the first member of the SRS family found in *Arabidopsis* [1]. The structure of the proteins encoded by the SRS transcription factor genes (SRSs) contains a conserved RING-like zinc-finger domain (CX2CX7CX4CX2C2X6C), and some proteins among them contain the IXGH domain [2]. The RING-like zinc-finger domain is one type of typical RING domain (C3H2C3 or C3HC4). The conserved RING domain was first found as a DNA-binding motif in animal *Xenopus laevis* [3]. This domain can bind to RNA, protein and lipid substrates, which showed that they possess multiple roles in many intracellular physiological and biochemical processes [4,5]. It is known that there is a cysteine-rich model coupling two zinc atoms in RING domain [6]. There are many types derived from typical RING domain, such as RING-V, RING-D, RING-S/T, RING-G and RING-C2 [6,7]. The IXGH domain contains acidic amino acids, which demonstrate that SRSs possessing IXGH domains are transcriptional activators [2,8].

In *Arabidopsis*, nine active members of the SHORT INTERNODES/STYLISH (SHI/STY) and SRS family were widely reported. The nine members possess both the RING-like zinc finger domain and IXGH domain. They play very important roles in the progress of plant growth. *LRP1* was involved in chromatin modification and auxin signaling during lateral root (LR) development by may forming a complex with SHI, STY1, SRS3, SRS6, and SRS7 [8,9,10]. Interestingly, *LRP1* as well as *STY1* could regulate the expression of *YUC4* in the process of auxin biosynthesis [8,11]. *STY1* (*SRS1*) was reported to regulate auxin biosynthesis, affect apical-basal patterning of stamen, influence cell expansion and timing of flowering [12,13]. *STY2* also promoted the formation of apical of stamen [14]. The mutant of *AtSHI* showed resembled phenotype of a mutant defective in gibberellin (GA) biosynthesis, demonstrating that *AtSHI* may be involved in the GA signaling pathway [15]. Overexpression of *AtSHI* in the *ornamental Kalanchoë* and the *poinsettia* caused compact phenotype of transgenic plants [16,17]. Interestingly, the members of the *SHI/STY* family had the redundant function in the progress of auxin biosynthesis probably by regulating the photomorphogenesis-related genes (*HY5*, *BBX21*, and *BBX22*) [11,13,18,19]. In addition to *Arabidopsis*, *LjSTY1/2/3* acted as the targets of LjNFYA1 which played an important role during nodule differentiation in *lotus japonicas* [20]. In rice, *OsSHI1* increased tiller number and diminished panicle size by modulating the transcriptional activity of IPA1 [21]. In barley, *LKS2* and *VRS2*, two members of SHI/STY family, regulated awn elongation, pistil morphology, and inflorescence patterning [22,23]. However, the genome-wide analysis and the function of this family in soybean are still not well characterized. 

Soybean is an important food and oil crop and its growth and productivity are easily susceptible to environmental stimuli [24,25]. Especially drought and salt stress have become one of the most abiotic stress problems for soybean development and growth [26,27,28,29,30,31]. One way to alleviate the yield loss is to discover the stress resistant genes to breed the robust soybean cultivar under the abiotic stress. In this study, we did a genome-wide analysis of soybean SRS family and investigated the potential functions in soybean response to various stimuli. We identified the function of *GmSRS18* in drought and salt responses. These results will give us a novel understanding about the soybean SRS family and provide the candidate resistant gene for soybean breeding.

## 2. Results

### 2.1. Identification of SRS Transcription Factors in Soybean

To excavate all the members of the SRS family, we searched the Plant Transcription Factor Database (http://planttfdb.cbi.pku.edu.cn/) and Phytozome v12.1 (https://phytozome.jgi.doe.gov/pz/portal.html) based on the conserved RING-like zinc-finger domain (DUF702). Moreover, we identified 21 members in soybean and 11 members in *Arabidopsis*. We analyzed the basal characteristic of 21 members in soybean containing amino acid residues (aa), molecular mass (KD), PI, chromosome, domain location, and the best hit in *Arabidopsis* (Table 1). We named them *GmSRS1*~*GmSRS21* based on their gene ID number. The number of amino acid residues (aa) ranges from 201 to 371, and the molecular mass (KD) ranges from 22323.7 to 41022.1. Interestingly, the characteristics of their proteins were mainly neutral and alkaline except GmSRS19 (PI 5.765). Meanwhile, we found their corresponding members with the highest homology in *Arabidopsis* (Table 1).

**Figure 1:**
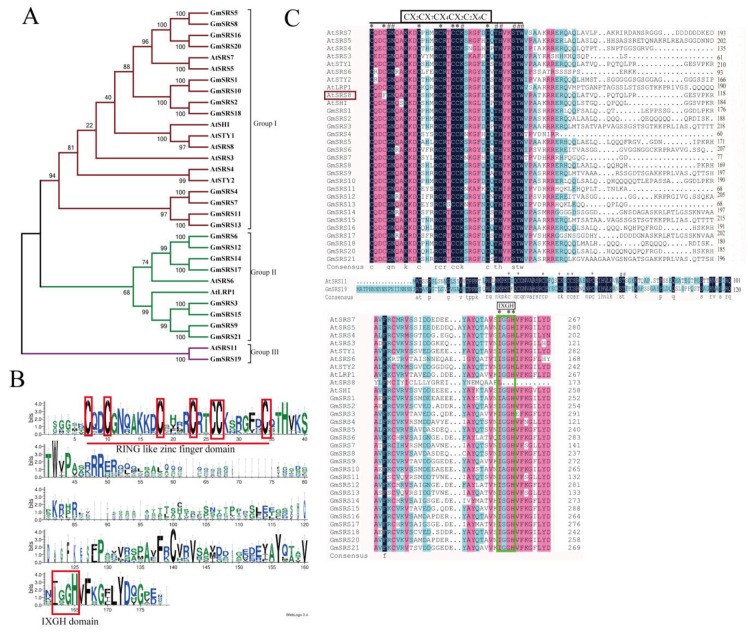
Phylogenetic relationship and predicted structures of SRS family proteins in soybean and *Arabidopsis*. (**A**) Neighbor-joining phylogenetic tree of the SRS family in soybean and *Arabidopsis*. (**B**) The conserved RING-like zinc finger domain (CX_2_CX_7_CX_4_CX_2_C_2_X_6_C) and the IXGH domain. The predicted domain sequence was obtained from the website PlantTFDB v5.0 (http://planttfdb.cbi.pku.edu.cn/) (**C**) Alignment of SRS proteins in soybean and *Arabidopsis*.

**Figure 2:**
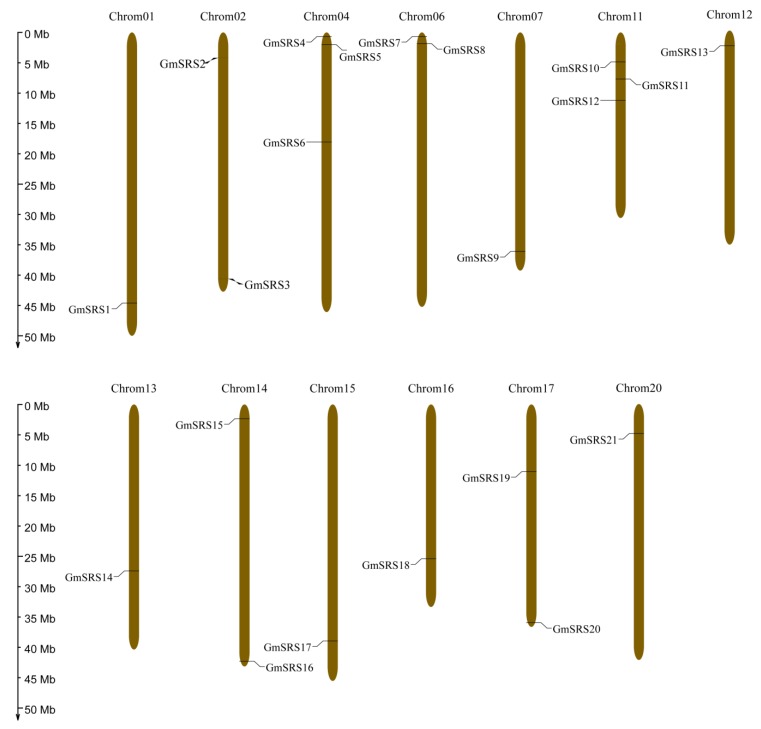
Chromosomal locations of soybean SRS genes. The ratio on the left represents the chromosome length.

To better realize the members of SRS family, we conducted the phylogenetic tree, which could easily evaluate the phylogenetic relationships of SRSs between soybean and *Arabidopsis*. We divided them into three groups (I, II, and III) (Figure 1A). In *Arabidopsis*, *AtSRS11* was in Group III, *AtLRP1* and *AtSRS6* in Group II, and others in Group I. The result was consistent with previous report that *AtLRP1* and *AtSRS6* had the highest homologous relationship [32]. We found that all members of this family contained the RING-like zinc finger domain (CX_2_CX_7_CX_4_CX_2_C_2_X_6_C) through sequence alignment, but the second cysteine residues of AtSRS8 mutate to phenylalanine residues (Figure 1B,C). Moreover, they had the IXGH domain except AtSRS11 and GmSRS19, which both belong to Group III (Figure 1C). The 21 members localized on 13 chromosomes in soybean (Figure 2).

### 2.2. Analysis of Gene Structure and Cis-Acting Elements

**Figure 3:**
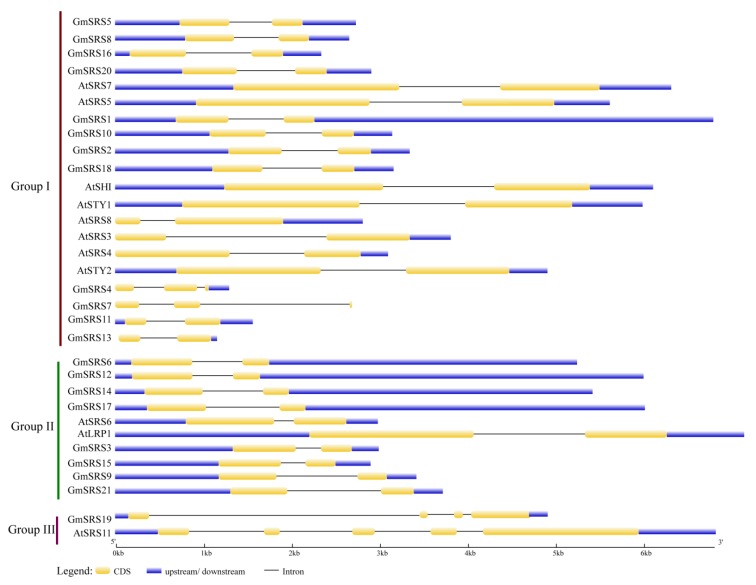
Intron-exon structures of *SRS* genes in soybean and *Arabidopsis*. The GSDS online tool was used to produce the intron-exon structures. The exons, introns, and untranslated regions (UTRs) are indicated by the white boxes, black lines, and gray line, respectively.

To better understand the structure, we drew the intron-exon model based on the genome sequence of soybean and *Arabidopsis* SRS genes. In Group I and II, they had two exons and one intron except *GmSRS4* and *Gm SRS7* which had three exons and two introns. In Group III, they had at least four exons and three introns (Figure 3). These may demonstrate that the members of Group I and II had the similar functions and *GmSRS19* and *AtSRS11* in Group III also had similar functions. 

The elements in the promoter of genes that can be recognized by transcription factors play crucial roles in progress of transcriptional control [33]. To predict the roles of SRSs in abiotic stress response, we analyzed the numbers of abiotic stress-related cis-elements, including ABA-responsive element (ABRE), E-BOX, GT-1, low-temperature responsive element (LTRE), drought responsive element MYB, and MYC, which were located on the promoters of SRS genes (Table 2). The promoters of SRS genes possess larger of ABRE, E-BOX, GT-1, MYB, and MYC elements. For example, every promoter had at least 16 GT-1, eight E-box, and eight MYC elements. Some promoters also had the temperature stress-related element LTRE. These results showed that the members may act as targets involving abiotic stress response in soybean.

### 2.3. Analysis of Expression Patterns in Various Tissues

**Figure 4:**
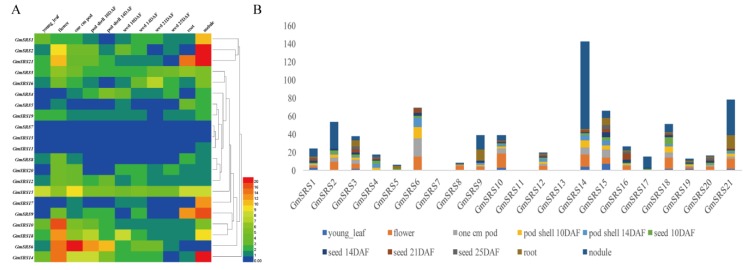
Expression patterns of soybean SRS genes in different tissues. The heat map (**A**) and histogram of soybean SRS genes expression patterns (**B**) in different tissues. The names are on the left and the tissue names are on the top of the figure, and the different colors indicate express degree of gene.

We searched the expression levels in 11 tissues containing young leaf, flower, 1 cm pod, pod shell 10 DAF, pod shell 14 DAF, seed 10 DAF, seed 14 DAF, seed 21 DAF, seed 25 DAF, root, and nodule based on the database SoyBase. The heat map shown that the members of SRS family expressed in leaf, flower, pod, pod shell, seed, root, and nodule. Except *GmSRS7* and *GmSRS13*, others remain higher expression levels in different tissues (Figure 4A). Especially, the expression levels of *GmSRS2*, *9*, *14*, and *21* were higher than that of others in nodule (Figure 4B). Moreover, *GmSRS6* was expressed primarily at the young leaf, flower, and pod, and *GmSRS9* was expressed at the root and nodule. Previous reports demonstrated the SRS family played vital roles in timing of flower [12,13]. *GmSRS2*, *3*, *6*, *8*, *9*, *10*, *12*, *14*, *15*, *16*, *18*, *20*, and *21* may all involved in the development of flower based on their expression in flower (Figure 4B) (Table S1). 

### 2.4. RNA-Seq Analysis of Drought-, NaCl-, and ABA-responsive SRS Genes

**Figure 5:**
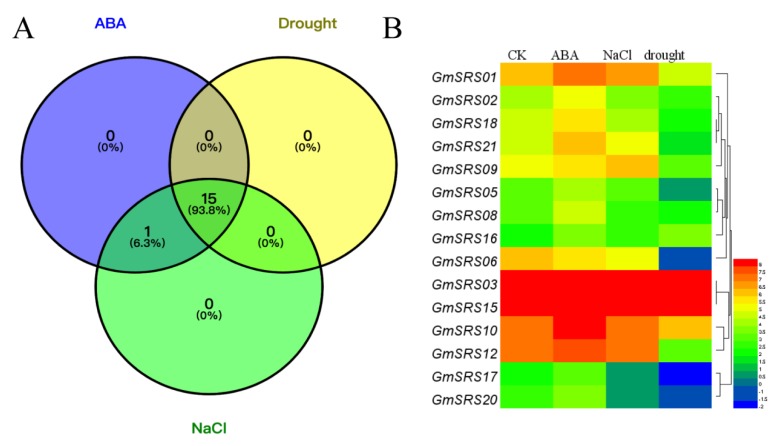
Transcriptome analysis-based soybean RNA sequencing data. (**A**) The Venn diagram and (**B**) the heatmap of stress-related genes under drought, NaCl, and abscisic acid (ABA) treatment.

To investigate the functions of the SRS family in the abiotic stress response, we performed soybean transcriptome sequencing analysis under drought, NaCl, and exogenous ABA treatment (Table S2). Fifteen members of SRS family in soybean were induced under drought, NaCl, and exogenous ABA treatment, and one member (*GmSRS11*) was only induced by exogenous ABA (Figure 5A). The expression of *GmSRS3* and *GmSRS15* were still maintaining a high level. Moreover, the expression of *GmSRS18* and *GmSRS21* was increased under ABA treatment and reduced under drought treatment (Figure 5A). *GmSRS6* was only negatively induced by drought and NaCl stresses. Although some members were induced by drought, NaCl, and exogenous ABA, the expression of them did not make a significant difference (Table S2).

### 2.5. Quantitative Real-Time PCR (qRT-PCR) Analysis of Soybean SRS Genes

**Figure 6:**
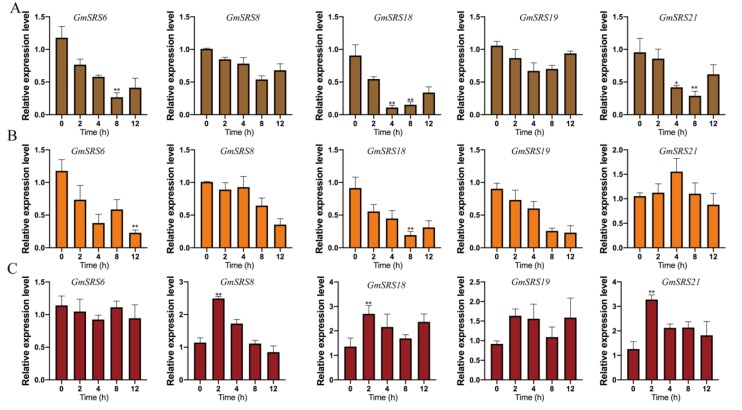
Relative expression levels of soybean *SRS* genes under drought, NaCl, and exogenous ABA treatment. quantitative Real-Time PCR (qRT-PCR) analyses of plants treated with drought (**A**), NaCl (**B**), and ABA (**C**). * *p* < 0.05, ** *p* < 0.01.

Phytohormone and environment stimuli severely affect crop growth and development. To investigate the potential roles of the soybean SRS genes in response to different stimuli, the expression patterns of these genes in soybean treated with ABA, NaCl, and drought were analyzed by qRT-PCR (Figure 6). We selected five members that were from Group I (*GmSRS8* and *GmSRS18*), Group II (*GmSRS6* and *GmSRS21*), and Group III (*GmSRS19*) for further analysis.

### 2.6. Subcellular Localization of GmSRS18 in Arabidopsis Protoplasts

**Figure 7:**
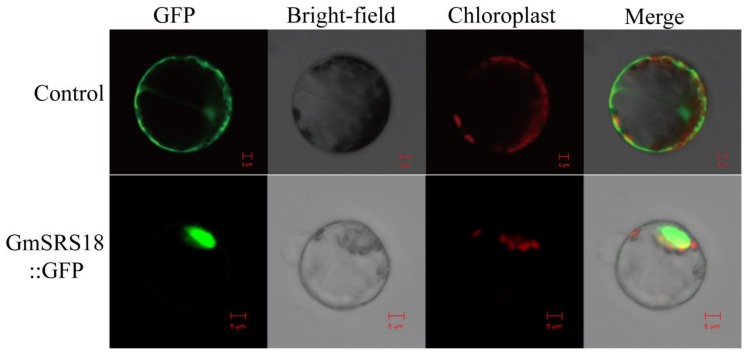
Subcellular localization of the soybean GmSRS18 protein. The picture above is a control and below is GmSRS18 protein. Results were visualized with confocal microscopy 16 h after transformation. Scale bars = 5 μm.

To investigate the functions of the SRS family, *GmSRS18,* which was from Group I and significantly induced by drought, NaCl, and ABA, was selected for further assay. Protein localization is closely related to its function [34,35]. The fused plasmid containing the full-length sequence of *GmSRS18* and the Green fluorescent protein (GFP) tag was transformed into *Arabidopsis* protoplasts. The empty vector 16318GFP as the control was transformed into *Arabidopsis* protoplasts. And the results showed that the control protein was localized in the whole cell including cell membrane, cell cytoplasm, and cell nuclear and the protein encoded by *GmSRS18* was only localized in cell nuclear (Figure 7), which suggested that GmSRS18 functioned mainly in the nucleus.

### 2.7. Overexpression of GmSRS18 Conferred Drought and Salt sensitivity in Arabidopsis

**Figure 8:**
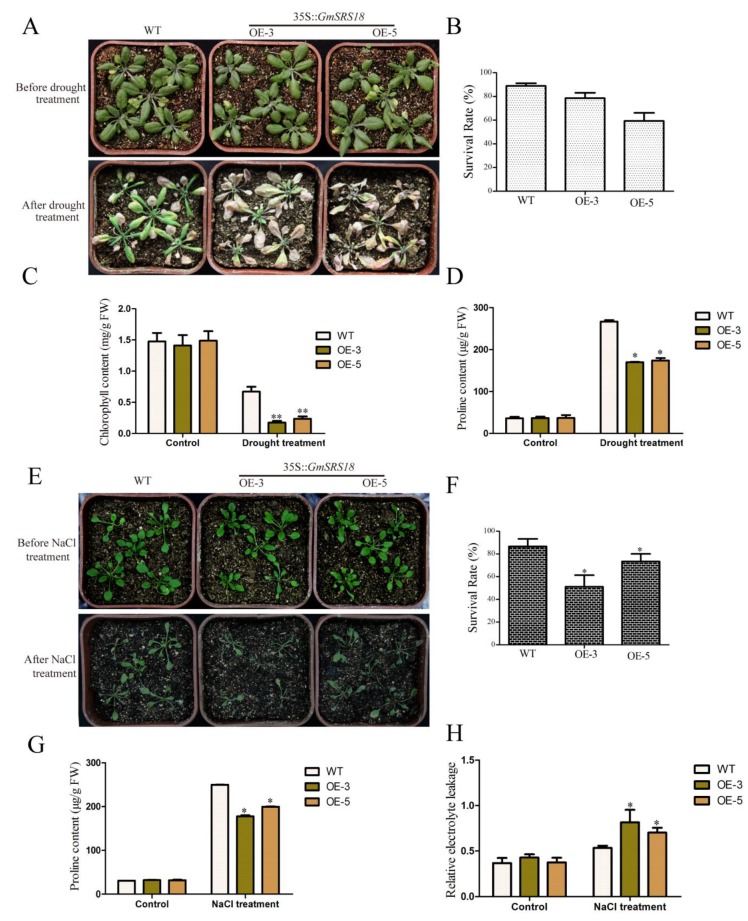
Phenotype of *GmSRS18* overexpression *Arabidopsis* under drought and salt treatment conditions. (**A**) The phenotype of wild type WT and transgenic lines (OE-3 and OE-5) under drought treatment. 15-day-old plants were subjected to drought stress. The drought related physiological index was measured, including survival rate (**B**), chlorophyll content (**C**), and proline content (**D**). (**E**) The phenotype of WT and transgenic lines under NaCl treatment. 10-day-old plants were subjected to NaCl stress. The salt stress-related physiological index was measured, including survival rate (**F**), proline content (**G**), and relative electrolyte leakage (**H**). The data are shown as mean ± Standard Deviation (SD) (*n* = 45). Independent t-tests demonstrated that there was significant difference (* *p* < 0.05, ** *p* < 0.01). Each experiment was repeated at least three times.

To investigate whether *GmSRS18* plays important role in regulating plant response to drought and salt stresses, we generated transgenic *Arabidopsis* overexpressing GmSRS18 in wild type (WT, Col-0). The expression level of *SRS18* in transgenic lines was investigated by qRT-PCR (Figure S1). Then, we selected two transgenic lines (OE-3 and OE-5) to identify their functions under drought and salt treatment. To examine the sensitivity of transgenic *Arabidopsis* to drought stress, 15-day-old seedlings containing WT and two transgenic lines were deprived water and the control lines were well watered. There was no significant difference among all lines when they were well watered. However, when they were deprived water, the seedlings of OE-3 and OE-5 lines exhibited more wilting and etiolated as compared with the WT seedlings (Figure 8A). To explain this difference visually, relevant physiological indexes were tested. The data analysis of the physiological indexes demonstrated that the two transgenic lines had a higher survival rate, lower chlorophyll content, and lower proline content than that of WT (Figure 8B–D). 

To further determine whether *GmSRS18* is also sensitive to salt, 10-day-old seedlings of WT and two transgenic lines were treated by NaCl solution. In the absence of NaCl, WT and transgenic lines exhibited similar phenotype. However, in the presence of NaCl, transgenic lines were more wilting than WT (Figure 8E). The lower survival rate, lower chlorophyll content and higher relative electrolyte leakage of transgenic lines showed that transgenic *Arabidopsis* were more sensitive to NaCl than WT (Figure 8F–H). These results suggested that *GmSRS18* was a negative regulator in the drought and salt stresses signaling pathway.

### 2.8. GmSRS18 Negatively Regulated Drought- and Salt-Related Gene Expression

**Figure 9:**
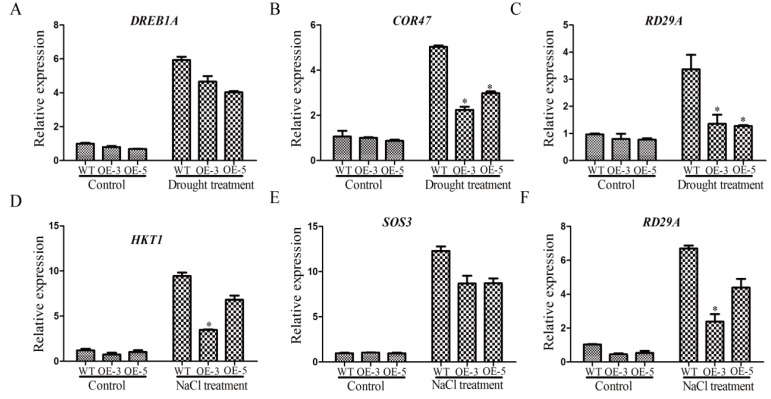
The expression levels of stress-related marker genes. Drought stress-related marker genes were tested, including DREB1A (**A**), COR47 (**B**), and RD29A (**C**). Salt stress-related marker genes were tested, including HKT1 (**D**), SOS3 (**E**), and RD29A (**F**). Independent t-tests demonstrated that there was a significant difference (* *p* < 0.05).

The expression levels of stress-related genes were affected by drought and salt stresses [36]. To try to explore the function mechanism of *GmSRS18*, we examined the expression changes of several stress-related marker genes, including *RD29A*, *DREB1A*, *COR47*, *HKT1*, and *SOS3*. Under normal conditions, there was no significant difference in their expression levels between WT and transgenic lines (Figure 9). Under drought treatment, compared with control plants, the transcript levels of *DREB1A*, *RD29A*, and *COR47* were lower in transgenic lines (Figure 9A–C). Likely, the transcript levels of *HKT1*, *RD29A*, and *SOS3* in transgenic lines were lower than that of WT. These results showed that *GmSRS18* may negatively regulate drought- and salt-related genes. 

## 3. Discussion

Transcription factors play vital roles in plant growth and development and many transcription factors have been identified to be involved in a series of progresses responding to abiotic and biotic stresses and signaling networks of plant hormones [37,38,39,40,41,42,43,44,45,46,47,48,49]. The annotation and structure analysis of many transcription factors in different plant species had been reported [50,51,52,53,54,55]. Ten members of SRS family were identified in *Arabidopsis* [1,14,15]. However, reports on the whole genome of SRS transcription factors family in soybean are relatively lacking. In this research, we first identified 21 members of SRS family in soybean and 11 members in *Arabidopsis*. Previous studies reported that SHI family also called SHI/STY family contained *SHI*, *STY1*, *STY2*, *LRP1*, *SRS3*, *SRS4*, *SRS5*, *SRS6*, *SRS7*, and *SRS8* in *Arabidopsis*. In addition to this, we referred to AtSRS11 (AT1G32730), which also had the conserved RING-like zinc finger domain, as one member of SRS family. In this way, we could define that the main characteristic of this family is that it contains a conserved RING-like zinc finger domain. Thus, the novel *Arabidopsis* SRS family contained previous SHI family and new member (SRS11) and was assigned to three groups (eight members in Group I, two members in Group II and one member in Group III). The phylogenetic analysis showed that SRS family in soybean was also divided into three groups based on their amino acid sequences (12 members in Group I, eight members in Group II, and one member in Group III) (Figure 1A). Major members of SRS family in soybean and *Arabidopsis* contain both the RING-like zinc finger domain and the IXGH domain, and some only contain the RING-like zinc finger domain, including the members in Group III and GmSRS8 in Group I (Figure 1C). We hypothesized that the IXGH domains in some members of SRS family were lost during evolution.

Introns are important structures affecting the rate of transcription, nuclear export, and transcript stability and increase the efficiency of mRNA translation [56,57,58]. Although introns could not function as a binding site for transcription factors, they can increase the gene expression [57]. We analyzed the gene structure and found that the members located in the same subfamily have similar gene structure, which proved a presumption that the members in same group had the similar evolutionary relationship or function (Figure 3). As described in the analysis of cis-acting elements in the promoter of SRS genes, they contained many stress-related elements, such as ABRE, E-BOX, GT-1, LTRE, MYB, and MYC (Table 2). ABRE-binding proteins (AREBs/ABFs) can bind to ABRE element to be involved in ABA, dehydration, and high-salinity stress responses [59]. In rice, OSBZ8, an ABRE-binding factor, may regulate the salt tolerance in *Indica* rice [60]. AtHSFA7b positively regulated the salinity tolerance in *Arabidopsis* through binding to the E-BOX motif [61]. The E-BOX motif acts as an important component in salinity tolerance response. The MYB element played vital roles in drought, salt, ABA, and GA responses [62,63]. Likely, MYC responds to drought, salt, and ABA stresses through combining with MYC binding proteins [64]. GT-1 may be related to pathogen and salt signals [65]. LTRE element is critical in responding to low temperature stress [66]. The members of the SRS family in soybean contained a number of stress-related elements, which demonstrated that they have a great relationship with the plant stress response. We also found that 15 members were simultaneously induced by ABA, NaCl, and drought, and one member was induced by ABA and NaCl based on the soybean transcriptome sequencing (Figure 5). The expression of the selected five members was inhibited under drought and NaCl treatment (Figure 6A,B). However, their expression increased under exogenous ABA treatment (Figure 6C). These indicated that *GmSRS18* regulated the drought and salt response by an ABA-dependent/independent pathway. 

*GmSRS18*, induced by drought, NaCl, and exogenous ABA, was selected for functional identification in *Arabidopsis*. GmSRS18, as a transcription factor, was located in the cell nucleus, which showed that it functions in cell nucleus. Transcription factors were involved in the abiotic and biotic stress response in many plants [67,68,69,70,71,72]. Though there are few reports of SRS family about the abiotic stress response thus far, we demonstrated that *GmSRS18* can increase the transgenic *Arabidopsis* sensitivity to drought and salt stresses. To further explain the function molecular mechanism of *GmSRS18*, the expression levels of drought- and salt-related marker genes were tested by qRT-PCR. *RD29A* was involved in drought and salt stresses response [73]. *COR47* and *DREB1A* were associated with drought stress and HKT1 and SOS3 were associated with salt stress [74,75,76,77,78,79,80]. They all were increased by drought and NaCl treatment. However, their transcript levels in transgenic *Arabidopsis* were lower than that of WT, which showed that they may be inhibited by *GmSRS18*. The relationship between *GmSRS18* and stress-related genes need further discussion and research, which can provide evidence for how GmSRSs can regulate drought and salt stresses response in plant. 

## 4. Materials and Methods

### 4.1. The Search of All Members in SRS Family in the Soybean and Arabidopsis

The database PlantTFDB v5.0 (http://planttfdb.cbi.pku.edu.cn/) and phytozome V12.1 (https://phytozome.jgi.doe.gov/pz/portal.html) (*Glycine max Wm82.a2.v1* and *Arabidopsis thaliana TAIR10*) were used to search all members in SRS family based on the conserved RING-like zinc-finger domain (DUF702) [81,82]. We then combined the data from PlantTFDB V5.0 and phytozome V12.1.

### 4.2. Phylogenetic Analysis and Alignment of SRSs Sequences 

The amino acid sequences of SRS members in soybean and *Arabidopsis* downloaded from database phytozome V12.1 were used to analysis by ClustalX (1.83) and MEGA7.0.21 [83]. The parameter of the neighbor-joining (NJ) algorithm in MEGA7.0.21 was set (No. of bootstrap replications: 1000). And the alignment of SRSs was conducted by DNAMAN V7 and the result calibration was done in Adobe Illustrator CS5 V15.0.0. 

### 4.3. Chromosome Locations of Soybean SRS Genes

We obtained the location information of SRS genes in soybean from the database phytozome V12.1. Moreover, we generated Chromosome locations using online tool MG2C v2.1 (http://mg2c.iask.in/mg2c_v2.1/). The picture calibration was done in Adobe Illustrator CS5 V15.0.0.

### 4.4. Gene Structure and cis-acting Element Analysis

The genome and CDS sequences of SRS genes in soybean and *Arabidopsis* download from phytozome V12.1 and the exon-intron substructure map was generated by the online tool Gene Structure Display Server 2.0 (GSDS http://gsds.cbi.pku.edu.cn/) [84]. The 2000 bp promoter before initial codon ATG was also download from phytozome V12.1 and was evaluated using Promoter 2.0 Prediction Server (http://www.cbs.dtu.dk/services/Promoter/) [85]. The cis-acting element were analyzed by the online tool NEW PLACE (https://www.dna.affrc.go.jp/PLACE/?action=newplace) [86].

### 4.5. The Expression Patterns Analysis of SRS Genes in Different Tissues

To analysis the expression of SRS genes in different tissues, we downloaded the Affimetrix soybean gene chip in SoyBase (http://www.soybase.org/soyseq/). Additionally, we generated the heat map containing expression levels in different tissues by using the software Heml 1.0 [87].

### 4.6. Plant Materials and Stress Treatments

Soybean *Tiefeng* 8 and *Arabidopsis Colombia*-0 (Col-0) were used in this research. Moreover, soybean seeds were grown in soil (nutrient soil: vermiculite 1:1) in a chamber at 25 °C with a 16 h light and 8 h dark photoperiod. Two-week-old seedlings were treated by various stimuli for Quantitative Real-time analysis. The treatment method was modified on the basis of previous reports [24]. For the drought treatment, the seedlings were transferred to vermiculite and dehydrated for 0, 2, 4, 8, and 12 h. For the NaCl treatment, the solution containing 200 mM NaCl was poured into the pot. For exogenous ABA treatment, the solution containing 100 μM ABA was sprayed on the soybean seedlings. The seedlings treated by drought, NaCl, and exogenous ABA were harvested at 0, 2, 4, 8, and 12 h. The detached samples were immediately thrown into liquid nitrogen, then, the samples were used to extract the RNA or stored at -80 °C refrigerator until RNA extraction. 

### 4.7. Soybean RNA-seq and Quantitative Real-time PCR (qRT-PCR)

The experimental methods of RNA-seq have been reported by our laboratory [88]. The leaves of 10-day-old soybean seedlings were treated by natural drought (5 h), 100 mM NaCl (1 h), and 100 μM exogenous ABA (1 h) for RNA-seq. We obtained all SRS genes from RNA-seq.

The total RNA was extracted from the treated sample following the instructions of the Fast plant RNApure Kit (ZOMANBIO, Beijing, China). The cDNA synthesis was conducted by using the kit (Transcript One-step gDNA Removal and cDNA Synthesis SuperMix) (TRANS, Beijing, China) as previously described [89]. The ABI Prism 7500 sequence detection system (Applied Biosystems, Foster City, CA, USA) was used to detect the expression patterns of SRS genes, and then, the data was dealt by the software Graphpad Prism 8 and was analyzed as previously described [85,86]. The primers used for qRT-PCR were designed by the software Primer Premier 5.0 (Table S3).

### 4.8. Subcellular Localization Analysis of GmSRS18 

The full-length CDS sequence of *GmSRS18* was cloned from soybean cDNA. Then, the CDS with BamH I enzyme site was ligated to the vector 16318GFP, which contained green fluorescent protein (GFP) tags and was controlled by the CaMV35S promoter. As described previously, the subcellular localization of *GmSRS18* and the control protein GFP were conducted by the *Arabidopsis* protoplasts. After 16 h of protoplast incubation, the sample was monitored by confocal microscopy (LSM700; CarlZeiss, Oberkochen, Germany) [85].

### 4.9. Functional analysis of GmSRS18 in Arabidopsis

To generate the transgenic *Arabidopsis*, the full-length CDS sequence of GmSRS18 was ligated into the pCAMBIA1302 vector under control of the CaMV 35S promoter. Referring to the previous *Arabidopsis* transformation method (floral dipping method), we generated the transgenic *Arabidopsis* [24]. The transgenic lines were selected by the antibiotics hygromycin (30 mg/L) and T3 generation lines (OE-3 and OE-5) were used as the following experimental material.

For drought resistant assessment, the 15-day-old seedlings of WT and transgenic lines were deprived water, and the control lines were well watered. After 10 days, we measured the survival rate, chlorophyll content, and proline content. For salt resistant ability, the 10-day-old seedlings of WT and transgenic lines were watered with 200 mM NaCl solution and the control lines were well watered. After four days, we measured the survival rate, relative electrolyte leakage, and proline content. 

### 4.10. Physiological Measurements

The chlorophyll and proline content were measured based on the method described previously with slight modification [90,91]. The leaf samples (0.1g) were transferred into the mix solution (50% alcohol and 50% acetone). The mixtures were incubated in darkness for 12 h, then centrifuged at 5000 *g* for 15 min at 4 °C. The instrument Varioskan Lux (Thermo scientific, Waltham, MA, USA) was used to photometrically quantified photometrically at 663 and 645 nm. The proline content was measured according to the method described previously [92]. The proline detecting kit (Comin, Beijing, China) provided the necessary reagents and instructions.

## Figures and Tables

**Table 1:** The characteristic information of the SHI-RELATED SEQUENCE (SRS) transcription factors.

Gene Name	Gene ID	Amino Acid Residues (aa)	Molecular Weight (MW) (Da)	P*I*	Chromosome	RING-LIKE Zinc Finger Domain	Bes Hit in *Arabidopsis*
*GmSRS1*	Glyma.01G170700	317	34290.8	6.5009	1	103~256	*STY1*
*GmSRS2*	Glyma.02G051900	329	35460.7	7.3971	2	115~259	*STY1*
*GmSRS3*	Glyma.02G280000	356	37500.2	8.3444	2	135~296	*LRP1*
*GmSRS4*	Glyma.04G009300	211	23087.5	7.9448	4	12~62	*SRS3*
*GmSRS5*	Glyma.04G027400	306	33185.7	6.8469	4	98~245	*SRS5*
*GmSRS6*	Glyma.04G136700	333	35377.4	8.1854	4	135~287	*LRP1*
*GmSRS7*	Glyma.06G009200	201	22323.7	8.4033	6	21~77	*SRS3*
*GmSRS8*	Glyma.06G027500	302	33032.5	7.7425	6	103~242	*SRS7*
*GmSRS9*	Glyma.07G230400	331	35295.6	7.0258	7	117~277	*LRP1*
*GmSRS10*	Glyma.11G072500	335	36133.9	6.6899	11	111~269	*SRS5*
*GmSRS11*	Glyma.11G113200	216	24179.3	9.1277	11	7~70	*SRS3*
*GmSRS12*	Glyma.11G155400	332	35606.7	8.5013	11	137~285	*LRP1*
*GmSRS13*	Glyma.12G039100	211	23370.3	8.5773	12	10~138	*SRS3*
*GmSRS14*	Glyma.13G197300	320	34173.9	8.0081	13	122~278	*LRP1*
*GmSRS15*	Glyma.14G034800	350	36900.8	8.3423	14	133~293	*LRP1*
*GmSRS16*	Glyma.14G216400	334	36073.6	7.2692	14	117~268	*SRS5*
*GmSRS17*	Glyma.15G235000	323	35013.6	7.7249	15	126~281	*LRP1*
*GmSRS18*	Glyma.16G132100	315	34541.7	7.1072	16	109~247	*SRS7*
*GmSRS19*	Glyma.17G150800	371	41022.1	5.765	17	45~81	*SRS11*
*GmSRS20*	Glyma.17G255000	327	35594.1	7.2575	17	111~262	*SRS5*
*GmSRS21*	Glyma.20G037100	341	36080.5	7.5825	20	113~274	*LRP1*

**Table 2:** The cis-acting elements in the promoters of soybean SRS genes.

Gene	ABRE	E-BOX	GT-1	LTRE	MYB	MYC
*GmSRS1*	10	8	16	2	4	10
*GmSRS2*	8	14	25	2	12	16
*GmSRS3*	2	10	35	0	3	14
*GmSRS4*	3	10	30	1	6	12
*GmSRS5*	6	16	27	1	8	18
*GmSRS6*	3	14	35	3	3	18
*GmSRS7*	2	18	39	1	2	22
*GmSRS8*	9	14	24	0	8	16
*GmSRS9*	6	18	16	1	7	26
*GmSRS10*	8	8	26	3	1	30
*GmSRS11*	1	8	25	0	4	14
*GmSRS12*	3	9	51	0	4	8
*GmSRS13*	5	14	36	0	6	14
*GmSRS14*	2	12	51	1	3	12
*GmSRS15*	2	14	35	1	5	18
*GmSRS16*	9	20	31	1	4	22
*GmSRS17*	5	8	35	0	4	8
*GmSRS18*	8	8	26	0	14	10
*GmSRS19*	4	8	27	0	4	8
*GmSRS20*	7	18	27	1	6	20
*GmSRS21*	1	10	37	1	4	16
Total	104	259	654	19	112	332

## Supplementary Materials

Supplementary materials can be found at https://www.mdpi.com/1422-0067/21/5/1810/s1.

## Abbreviations

ABA	abscisic acid
qRT-PCR	quantitative Real-Time PCR
WT	wild type
ABRE	ABA-responsive element
GFP	Green fluorescent protein
