# Supplementary material for: Genome-Wide Analysis of the Shi-Related Sequence Family and Functional Identification of GmSRS18 Involving in Drought and Salt Stresses in Soybean
Source: Int J Mol Sci. 2020 Mar 6;21(5):1810. doi: 10.3390/ijms21051810 (PMC7084930; doi:10.3390/ijms21051810)
Supplement: Supplementary file 1 [file ijms-21-01810-s001.zip › Figure S1.docx]

**Figure S1.** The relative expression level of *GmSRS18* in transgenic lines.
